# Supplementary material for: An Update on the Surveillance of Livestock Diseases and Antimicrobial Use in Sierra Leone in 2021—An Operational Research Study
Source: Int J Environ Res Public Health. 2022 Apr 27;19(9):5294. doi: 10.3390/ijerph19095294 (PMC9104805; doi:10.3390/ijerph19095294)
Supplement: Supplementary file 1 [file ijerph-19-05294-s001.zip › ijerph-1632917-supplementary.pdf]

Table S1. Number of livestock treated with antimicrobials or anthelmintic drugs use by region and livestock species in Sierra Leone, March- October 2021[1]

Region	Livestock species	Susceptible livestock	Sick animals		Livestock treated with antimicrobials		Livestock treated with anthelmintics	
		n	n	(%)[2]	n	(%)[3]	n	(%)[3]
Northern	Cattle	888	294	(33.1)	153	(52.0)	135	(45.9)
	Dogs	63	6	(9.5)	0	(0.0)	4	(66.7)
	Donkeys	3	1	(33.3)	1	(100.0)	0	(0.0)
	Fowl	594	150	(25.3)	0	(0.0)	3	(2.0)
	Goat	4483	2052	(45.8)	608	(29.6)	561	(27.3)
	Goats and Sheep[4]	1293	804	(62.2)	382	(47.5)	378	(47.0)
	Horse	37	15	(40.5)	15	(100.0)	15	(100.0)
	Pig	1472	345	(23.4)	19	(5.5)	60	(17.4)
	Rabbit	0	0	(0)	0	(0.0)	0	(0.0)
	Sheep	1135	416	(36.7)	131	(31.5)	291	(70.0)
North West	Cattle	260	62	(23.8)	9	(14.5)	49	(79.0)
	Dogs	8	2	(25.0)	1	(50.0)	1	(50.0)
	Donkeys	0	0	(0)		(0.0)	0	(0.0)
	Fowl	1334	212	(15.9)	3	(1.4)	40	(18.9)
	Goat	2322	696	(30.0)	377	(54.2)	354	(50.9)
	Goats and Sheep[4]	0	0	(0)	0	(0.0)	0	(0.0)
	Horse	0	0	(0)	0	(0.0)	0	(0.0)
	Pig	40	15	(37.5)	0	(0.0)	0	(0.0)
	Rabbit	40	18	(45.0)	0	(0.0)	11	(61.1)
	Sheep	1408	320	(22.7)	164	(51.3)	183	(57.2)
Western	Cattle	0	0	(0)	0	(0.0)	0	(0.0)
	Dogs	86	70	(81.4)	37	(52.9)	21	(30.0)
	Donkeys	4	3	(75.0)	0	(0.0)	0	(0.0)
	Fowl	0	0	(0)	0	(0.0)	0	(0.0)
	Goat	374	220	(58.8)	95	(43.2)	119	(54.1)
	Goats and Sheep[4]	0	0	(0)	0	(0.0)	0	(0.0)
	Horse	3	1	(33.3)	0	(0.0)	1	(100.0)
	Pig	51	31	(60.8)	5	(16.1)	0	(0.0)
	Rabbit	12	12	(100.0)	4	(33.3)	12	(100.0)
	Sheep	228	115	(50.4)	63	(54.8)	56	(48.7)
Eastern	Cattle	17	5	(29.4)	5	(100.0)	5	(100.0)
	Dogs	533	27	(5.1)	5	(18.5)	10	(37.0)
	Donkeys	0	0	(0)	0	(0.0)	0	(0.0)
	Fowl	673	146	(21.7)	0	(0.0)	0	(0.0)
	Goat	3486	988	(28.3)	352	(35.6)	506	(51.2)
	Goats and Sheep[4]	36	8	(22.2)	0	(0.0)	8	(100.0)
	Horse	0	0	(0)	0	(0.0)	0	(0.0)
	Pig	152	107	(70.4)	76	(71.0)	90	(84.1)
	Rabbit	0	0	(0)	0	(0.0)	0	(0.0)
	Sheep	1540	487	(31.6)	225	(46.2)	258	(53.0)

Southern	Cattle	10	1	(10.0)	1	(100.0)	0	(0.0)
	Dogs	21	12	(57.1)	0	(0.0)	2	(16.7)
	Donkeys	0	0	(0)	0	(0.0)	0	(0.0)
	Fowl	3968	406	(10.2)	0	(0.0)	17	(4.2)
	Goat	11533	2273	(19.7)	144	(6.3)	496	(21.8)
	Goats and Sheep[4]	80	23	(28.8)	20	(87.0)	13	(56.5)
	Horse	0	0	(0)	0	(0.0)	0	(0.0)
	Pig	215	76	(35.3)	5	(6.6)	50	(65.8)
	Rabbit	0	0	(0)	0	(0.0)	0	(0.0)
	Sheep	6464	1353	(20.9)	109	(8.0)	203	(14.8)

[1] Reports with discrepancies between numbers of susceptible vs sick vs treated were excluded from analysis

[2] Percentage of sick out of susceptible animals

[3] Percentage of animals initiated on antimicrobial or anthelmintic treatment out of sick animals

[4]Some reports mentioned species as "Goats and sheep", and disaggregated species wise information was not available, hence retained as a separate category

Table S2. Antimicrobials commonly used in animal species and their classification as per standard classification systems

Name of antimicrobial	OIE classification of antimicrobials for veterinary use	WHO critically important antimicrobials for human medicine categories	European Medicines Agency classification	AwaRe classification
Tylosin	VCI	CI (Highest priority)	C	Not listed
Oxytetracycline	VCI	HI	D	W
Trimethoprim-Sulphamethoxazole	VCI	HI	D	A
Penicillin	VCI	HI	D	A
Gentamicin	VCI	CI (High priority)	C	A
Metronidazole	Not listed	I	D	A
Ciprofloxacin	VCI	CI (Highest priority)	B	W
Levofloxacin	VCI	CI (Highest priority)	B	W
Nozomil	Not listed	Not listed	Not listed	Not listed

VCI- Veterinary Critically Important Antimicrobial Agents, CI- Critically Important, HI- Highly Important, I-Critically Important, B-Restrict, C-Caution, D-Prudence, A-Access, W-Watch

Table S3. Antimicrobial use based on European Medicines Agency and AwaRe classifiation[1] among livestock in Sierra Leone, March- October 2021[2]

**Livestock species**	**Number of livestock treated with antimicrobials**	**European Medicines Agency classification**						**AwaRe classification**			
		**Restrict**		**Caution**		**Prudence**		**Access**		**Watch**	
	**n**	**n**	**(%)[3]**	**n**	**(%)[3]**	**n**	**(%)[3]**	**n**	**(%)[3]**	**n**	**(%)[3]**
Cattle	168	0	(0.0)	30	(17.9)	115	(68.5)	37	(22.0)	78	(46.4)
Dogs	43	17	(39.5)	0	(0.0)	20	(46.5)	2	(4.7)	35	(81.4)
Donkeys	1	0	(0.0)	0	(0.0)	0	(0.0)	0	(0.0)	0	(0.0)
Fowl	3	0	(0.0)	0	(0.0)	3	(100.0)	0	(0.0)	3	(100.0)
Goat	1576	16	(1.0)	274	(17.4)	785	(49.8)	43	(2.7)	775	(49.2)
Goats and Sheep[4]	402	0	(0.0)	31	(7.7)	371	(92.3)	46	(11.4)	335	(83.3)
Horse	15	0	(0.0)	0	(0.0)	15	(100.0)	0	(0.0)	15	(100.0)
Pig	105	0	(0.0)	8	(7.6)	81	(77.1)	0	(0.0)	81	(77.1)
Rabbit	4	0	(0.0)	0	(0.0)	4	(100.0)	0	(0.0)	4	(100.0)
Sheep	692	11	(1.6)	131	(18.9)	418	(60.4)	0	(3.9)	418	(60.4)
Not recorded	11	0	(0.0)	0	(0.0)	2	(18.8)	0	(0.0)	2	(18.8)
Total	3020	44	(1.4)	474	(15.6)	1814	(60.1)	155	(5.1)	1734	(57.4)

[1] A-Access, W-Watch, Re-Reserve

[2]Reports with discrepancies between numbers of susceptible vs sick vs treated, Multiple antimicrobials belonging to different categories may be used for the same condition,

[3]Percentages calculated out of all animals initiated on antimicrobial treatment

[4]Some reports mentioned species as "Goats and sheep", and disaggregated species wise information was not available, hence retained as a separate category

Table S4. Number of animals that were initiated on specific antimicrobials based on species in Sierra-Leonne, March-October 2021

**Animal species**	**Number of animals treated with antimicrobials**	**Tylosin**		**Oxytetracycline**		**Trimethoprim sulphamethoxazole**		**Gentamicin**		**Penicillin**		**Nozomil**		**Metronidazole**		**Ciprofloxacin**		**Levofloxacin**		**No Antibiotic specified**	
		n	(%)[2]	n	(%)[2]	n	(%)[2]	n	(%)[2]	n	(%)[2]	n	(%)[2]	n	(%)[2]	n	(%)[2]	n	(%)[2]	n	(%)[2]
Cattle	168	0	(0.0)	78	(46.4)	5	(3.0)	0	(0.0)	32	(19.0)	0	(0.0)	0	(0.0)	0	(0.0)	0	(0.0)	42	(25.0)
Dogs	43	30	(69.8)	18	(41.9)	2	(4.7)	0	(0.0)	0	(0.0)	4	(9.3)	0	(0.0)	14	(32.6)	3	(7.0)	8	(18.6)
Donkeys	1	0	(0.0)	0	(0.0)	0	(0.0)	0	(0.0)	0	(0.0)	0	(0.0)	0	(0.0)	0	(0.0)	0	(0.0)	0	(0.0)
Fowl	3	0	(0.0)	3	(100.0)	0	(0.0)	0	(0.0)	0	(0.0)	0	(0.0)	0	(0.0)	0	(0.0)	0	(0.0)	0	(0.0)
Goat	1576	271	(17.2)	759	(48.2)	26	(1.6)	13	(0.8)	0	(0.0)	0	(0.0)	4	(0.3)	0	(0.0)	16	(1.0)	659	(41.8)
Goats and Sheep[4]	402	21	(5.2)	335	(83.3)	0	(0.0)	10	(2.5)	36	(9.0)	0	(0.0)	0	(0.0)	0	(0.0)	0	(0.0)	7	(1.7)
Horse	15	0	(0.0)	15	(100.0)	0	(0.0)	0	(0.0)	0	(0.0)	0	(0.0)	0	(0.0)	0	(0.0)	0	(0.0)	0	(0.0)
Pig	105	8	(7.6)	81	(77.1)	0	(0.0)	0	(0.0)	0	(0.0)	0	(0.0)	0	(0.0)	0	(0.0)	0	(0.0)	16	(15.2)
Rabbit	4	0	(0.0)	4	(100.0)	0	(0.0)	0	(0.0)	0	(0.0)	0	(0.0)	0	(0.0)	0	(0.0)	0	(0.0)	4	(100.0)
Sheep	692	160	(23.1)	397	(57.4)	20	(2.9)	4	(0.6)	0	(0.0)	0	(0.0)	3	(0.4)	2	(0.3)	9	(1.3)	223	(32.2)
Not recorded	11	1	(9.1)	2	(18.2)	0	(0.0)	0	(0.0)	0	(0.0)	0	(0.0)	0	(0.0)	0	(0.0)	0	(0.0)	8	(72.7)
Total	3020	491	(16.3)	1692	(56.0)	53	(1.8)	27	(0.9)	68	(2.3)	4	(0.1)	7	(0.2)	16	(0.5)	28	(0.9)	967	(32.0)

[1]Reports with discrepancies between numbers of susceptible vs sick vs treated were excluded from analysis

[2] Percentage of sick out of susceptible animals

[3] Percentage of animals initiated on antimicrobial or anthelmintic treatment out of sick animals

[4]Some reports mentioned species as “Goats and sheep” and disaggregated species wise information was not available, hence retained as a separate category
